# Unanesthetized Rodents Demonstrate Insensitivity of QT Interval and Ventricular Refractory Period to Pacing Cycle Length

**DOI:** 10.3389/fphys.2018.00897

**Published:** 2018-07-11

**Authors:** Wesam Mulla, Roni Gillis, Michael Murninkas, Hadar Klapper-Goldstein, Hovav Gabay, Michal Mor, Sigal Elyagon, Noah Liel-Cohen, Olivier Bernus, Yoram Etzion

**Affiliations:** ^1^Cardiac Arrhythmia Research Laboratory, Department of Physiology and Cell Biology, Faculty of Health Sciences, Ben-Gurion University of the Negev, Beer-Sheva, Israel; ^2^Regenerative Medicine and Stem Cell Research Center, Ben-Gurion University of the Negev, Beer-Sheva, Israel; ^3^Cardiology Department, Soroka University Medical Center and the Faculty of Health Sciences, Ben-Gurion University of the Negev, Beer-Sheva, Israel; ^4^L’Institut de Rythmologie et Modélisation Cardiaque, l’Institut Hospitalo-Universitaire, Fondation Bordeaux Université, Bordeaux, France

**Keywords:** ECG, effective refractory period, monophasic action potential, rate-adaptation, rodent cardiac electrophysiology, QT interval

## Abstract

**Aim:** The cardiac electrophysiology of mice and rats has been analyzed extensively, often in the context of pathological manipulations. However, the effects of beating rate on the basic electrical properties of the rodent heart remain unclear. Due to technical challenges, reported electrophysiological studies in rodents are mainly from *ex vivo* preparations or under deep anesthesia, conditions that might be quite far from the normal physiological state. The aim of the current study was to characterize the ventricular rate-adaptation properties of unanesthetized rats and mice.

**Methods:** An implanted device was chronically implanted in rodents for atrial or ventricular pacing studies. Following recovery from surgery, QT interval was evaluated in rodents exposed to atrial pacing at various frequencies. In addition, the frequency dependence of ventricular refractoriness was tested by conventional ventricular programmed stimulation protocols.

**Results:** Our findings indicate total absence of conventional rate-adaptation properties for both QT interval and ventricular refractoriness. Using monophasic action potential recordings in isolated mice hearts we could confirm the previously reported shortening of the action potential duration at fast pacing rates. However, we found that this mild shortening did not result in similar decrease of ventricular refractory period.

**Conclusion:** Our findings indicate that unanesthetized rodents exhibit flat QT interval and ventricular refractory period rate-dependence. This data argue against empirical use of QT interval correction methods in rodent studies. Our new methodology allowing atrial and ventricular pacing of unanesthetized freely moving rodents may facilitate more appropriate utility of these important animal models in the context of cardiac electrophysiology studies.

## Introduction

Mice and rats are used extensively in cardiac research to study pathophysiological mechanisms of human diseases (Gill et al., 1989; Lin et al., 1995; London, 2001; Jacob and Kwitek, 2002; Tarnavski et al., 2004; Bhindi et al., 2006). While mice have clear advantages for the development of genetically engineered models, rats have been widely used for translational research due to a wealth of phenotypic data and its physiological similarities to humans (Huang et al., 2011). Despite the usefulness in cardiac research including modeling of cardiac arrhythmias, rodents possesses some electrophysiological characteristics that limit a direct comparison of the rodent and human electrocardiogram (ECG) (Boukens et al., 2014; Konopelski and Ufnal, 2016). In order to allow extrapolation of insights gained from rodent models to the human conditions, knowledge of the similarities and differences between the rodent and human cardiac electrophysiology is of crucial importance (Farraj et al., 2011; Kaese and Verheule, 2012; Boukens et al., 2014; Konopelski and Ufnal, 2016).

One of the parameters routinely assessed in the ECG is the QT interval (Redfern et al., 2003; Drew et al., 2010; Kaese et al., 2013). It is defined as the interval from the onset of the QRS complex, that is, the earliest indication of ventricular depolarization, to the end of the T wave, that is, the latest indication of ventricular repolarization (Rautaharju et al., 2009). Congenital or acquired heart diseases that affect cardiac repolarization and produce altered ventricular action potentials manifest on the ECG as prolonged or short QT intervals (Viskin, 2009). Clinical manifestations include a tendency to develop syncope or cardiac arrest due to spontaneous polymorphic ventricular arrhythmias (Postema and Wilde, 2014). It is well-known that QT intervals in humans are dependent on the heart rate (HR) and need to be corrected for HR to improve risk stratification or to standardize QT intervals in experimental subjects (De Bruyne et al., 1999; Rautaharju et al., 2009; Holzgrefe et al., 2014). Clinical studies of atrial pacing have provided direct evidence that changes in HR alone without autonomic modulation affect the QT interval as a consequence of intrinsic myocardium properties in humans (Ahnve and Vallin, 1982; Arnold et al., 1982; Franz et al., 1988; Zabel et al., 2000; Seethala et al., 2011). This holds true also for experimental models in primates, dogs, cats, rabbits, and guinea pigs (Janse et al., 1969; Elzinga et al., 1981; Hayes et al., 1994; Sakaguchi et al., 2009; Morissette et al., 2015). However, in rats and mice the relation of the QT duration to HR is debatable (Hayes et al., 1994; Ohtani et al., 1997; Mitchell et al., 1998; Tavakoli et al., 2007; Kmecova and Klimas, 2010; Speerschneider and Thomsen, 2013; Roussel et al., 2016). For example, Mitchell et al. (1998) used the natural daily variation in RR intervals in unanesthetized mice and found a strong correlation between the QT interval and HR, where slower HRs were associated with longer QT intervals. In contrast, Roussel et al. (2016) recently showed by using the same natural daily variation in RR intervals and after dosing mice with tachycardic agents (norepinephrine or nitroprusside) that increased HR was not associated with apparent shortening of the QT interval. In addition, correcting the QT intervals with Mitchell’s formula in Roussel et al. (2016), introduced an apparent dependence of the corrected QT (QTc) on the HR. This dependence, which was noted with the most commonly used QT correction method in mice, is contrary to the expected adaptation of the QT to the stimulation frequency. In rats, a QT correction formula suggested by Kmecova and Klimas (2010) was validated using pharmacological manipulations affecting HR. Interestingly, while drugs with multiple effects (e.g., isoproterenol) suggested dependence of the QT on the beating rate, the QT interval remained unaltered following the application of ivabradine, which selectively affect the HR alone.

Conflicting data also exist regarding the rate-adaptation properties of action potential duration (APD) and effective refractory period in the mice myocardium. In large mammals APD and effective refractory period consistently become shorter at fast pacing rates (Franz et al., 1988). While monophasic action potential (MAP) measurements in both atrial and ventricular preparations from mice indicated similar shortening of APD_90_ at fast pacing rates (Knollmann et al., 2007; Waldeyer et al., 2009), we and others have demonstrated flat rate-adaptation curves of atrial and ventricular refractoriness using conventional S1S2 pacing protocols in the mice heart (Etzion et al., 2008; Waldeyer et al., 2009). In contrast to mice, recordings in rat preparations typically do not demonstrate shortening of APD_90_ at fast pacing rates (Benoist et al., 2011, 2012). However, APD restitution has been observed in a rat model of pulmonary hypertension and was recently implicated in the induction of negative mechanical restitution in this model (Hardy et al., 2018).

Chronic implantation of cardiac pacing electrodes is technically challenging in rodents. As a result, reported pacing studies were mainly performed in *ex vivo* preparations or under deep anesthesia, conditions that might be quite far from the normal physiological state. Our laboratory developed an instrumented device that facilitates atrial and ventricular pacing studies in unanesthetized rodents (Etzion et al., 2008; Mor et al., 2014; Mulla et al., 2017). In this study, we took advantage of this methodology and explored in detail the rate-adaptation properties of freely moving rats and mice. Our findings indicate total absence of conventional rate-adaptation of the QT interval and ventricular effective refractory period (VERP) in both species. In practical terms our findings support the notion that the QT interval should not be corrected for HR in rodent studies and thus indicate that the non-corrected QT interval gives the most direct insight into experimental changes of ventricular repolarization in rodents.

## Materials and Methods

The study was carried out in strict accordance with the Guide for the Care and Use of Laboratory Animals of the National Institutes of Health. All animal studies reported in this article were approved by the institutional ethics committee of Ben-Gurion University of the Negev, Israel. Adult male C57BL6 mice (25–30 g) and Sprague-Dawley rats (250–320 g) were obtained from Harlan Laboratories (Rehovot, Israel). The animals were kept in separated rooms under standardized conditions throughout the study, according to home office guidelines: 12:12 light:dark cycles at 20–24°C and 30–70% relative humidity. Animals were free-fed autoclaved rodent chow and had free access to reverse osmosis filtered water. Following the electrophysiological studies described here most of the animals were utilized for long-term atrial or ventricular pacing studies, which are beyond the scope of this report (all approved by the ethics committee of Ben-Gurion University of the Negev, Israel). At the end of all experiments animals were euthanized under deep anesthesia.

### Rodent Pacing Device and Its Surgical Implantation

The rat implantable device and the surgical technique for permanent implantation of one or two bipolar-electrodes in predetermined epicardial locations (atrial or ventricular) were previously described in detail (Etzion et al., 2008; Mor et al., 2014; Mulla et al., 2017). For mice the device was similar in its general design, but substantially smaller. The weight of the device for mice did not exceed 1.5 g. For both devices the electrical connection to the epicardial surface was obtained using the miniature-bipolar hook electrode (MBHE) that was designed by our group for various *in vivo* electrophysiological applications in rodents (Etzion et al., 2008). Each MBHE contains a distal head with two sharp tungsten pins that are curved and isolated by insulated coating up to their tips. By means of small lateral thoracotomy the MBHE can be fixed on selected epicardial locations without the need for additional suturing. The implantable device is composed of an 8 pin ‘female’ connector that is attached by highly flexible insulated electrical wires (AS155–36, Conner Wire, Chatsworth, CA, United States and A-M Systems, Carlsborg, WA, United States; coated diameter 140–200 μm for rats and mice, respectively) to each MBHE. Additional electrodes for peripheral ECG like measurements (pseudo-ECG) were implanted subcutaneously. The peripheral leads included two poles that were located in the subcutaneous tissue in close proximity to each of the upper limbs and a third electrode that was implanted caudally in the back, in close proximity with the lower limbs and the base of the tail. Electron beam radiation is applied for sterilization of the device before its use. For device implantation animals were anesthetized (IP ketamine/xylazine 75/5 and 120/10 mg kg^-1^ for rats and mice, respectively) and mechanically ventilated using Inspira and Minivent respirators (Harvard Apparatus, Boston, MA, United States) for rats or mice, respectively. The animals were placed on a warmed heating pad and under sterile conditions the MBHEs were implanted on the desired sites. In mice, electrode implantation was performed using binocular magnification. Following chest closure the 8-pin ‘female’ connector was exteriorized through the skin of the back. Post-operative recovery and analgesia were performed as described previously. Buprenorphine (0.1 mg kg^-1^) was administrated by subcutaneous injection during immediate recovery and every 24 h during the first 3 days. In addition, Dipyrone (0.8 mg ml^-1^) was added to the drinking water during the first 3 days. The animals were monitored on a daily basis for signs of stress or inappropriate weight loss, according to guidance from the Ben-Gurion University veterinary services [assured by the Office of Laboratory Animal Welfare, United States (OWLA) #A5060-01, and fully accredited by the Association for Assessment and Accreditation of Laboratory Animal Care International (AAALAC)].

### Experimental Design

Rate adaptation properties of the unanesthetized rodent heart were evaluated in 38 implanted animals (Rats *n* = 20, Mice *n* = 18). QT intervals were measured in animals implanted with right atria (RA) MBHE (Rats *n* = 10, Mice *n* = 9). VERP was measured in animals implanted with left ventricle (LV) MBHE (Rats *n* = 10, Mice *n* = 9). Additional isolated mice hearts (*n* = 5) were used for ventricular MAP recordings in the LV to evaluate simultaneously the APD in comparison with the VERP.

### Electrophysiological Measurements in Unanesthetized Rats and Mice

Six days after implantation of the pacing device animals were placed in dedicated pacing and recording cages in which the electrical connector in their back was linked by an elastic cable to the pacing and recording apparatus. Pacing was performed using optically isolated units (STG4002-16mA, Multichannels, Reutlingen, Germany) at twice-diastolic threshold, using bipolar symmetrical pulses of 2-ms duration in each polarity (constant current mode). Pseudo ECG signals were recorded using a conventional amplifier (Model 1700, A-M Systems, Carlsborg, WA, United States). In each animal, we figured the lead configuration in which the T wave was most clearly observed. This was typically obtained using a differential recording between the two leads near the upper limbs while the caudal lead was connected to the ground. This configuration is most similar to a conventional lead II configuration of the ECG. However, the exact location of each peripheral lead was not totally uniform. Thus, we use the term Pseudo-ECG throughout the paper to indicate the undetermined vectors of this peripheral signal. Electrophysiological signals were interfaced with a PC using an A/D converter (PCI-6024E; National Instruments, Austin, TX, United States) and a program developed by YE (using LabView 7.1, National Instruments) to control data acquisition, electrical stimulation, and offline analysis.

**FIGURE 1:**
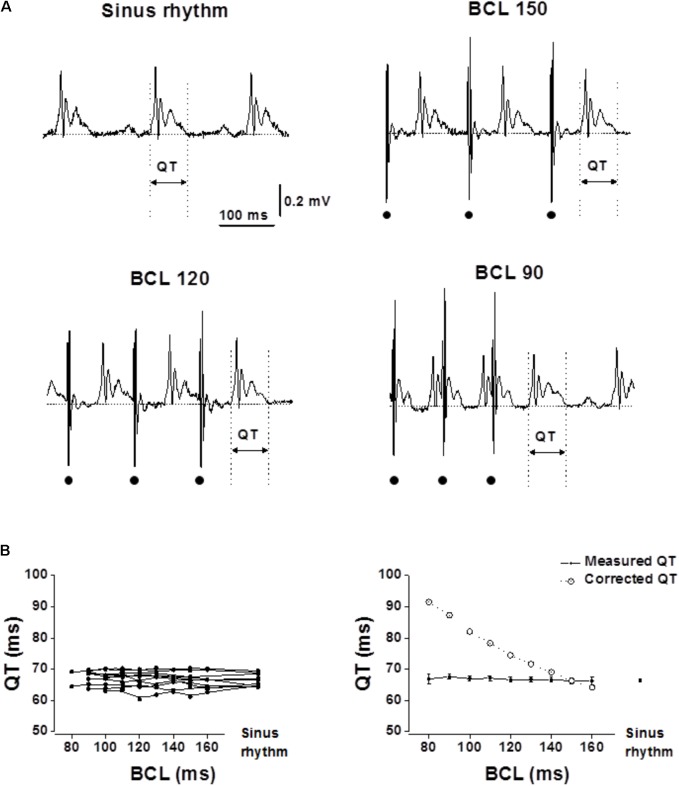
Measured QT interval in unanesthetized rats show flat rate dependence. Analysis of the QT interval in rats following atrial pacing at various BCLs. RA pacing was performed with an implanted MBHE on the RA. **(A)** Example of ECG recordings at sinus rhythm and during atrial pacing at various BCLs (150, 120, and 90). Dots at the bottom indicate atrial pacing. **(B)** Left: QT interval measurements of individual rats as a function of BCL and during sinus rhythm (*n* = 10). Right: Averages of measured QT and calculated QTc plotted as a function of BCL. Statistical analysis did not reveal any significant changes in QT measurement as a function of BCL. For detailed data of individual rats see **Supplementary Table S1**.

The animals were allowed to adapt for 24 h in the cage. On the 7th post-operative day, ECG was recorded at sinus rhythm and the relevant electrophysiological study was performed. For QT evaluation at various frequencies 20 beats of RA pacing were applied at a range cycle length (CL) starting from near normal and decreased by 10 ms steps until the 1:1 ventricular response was lost. Since at the shorter range of CL the stimulus artifact typically distorted the QT signal of the previous cycle, we applied for each CL 10 drive trains of twenty beats. In each of the drive train we analyzed only the last QT interval that was not distorted by an artifact and averaged the obtained value of the 10 drive trains in each CL (see **Figure 1A**, BCL 120 and BCL 90 as examples). The QT interval was measured between the first deviation from the isoelectric PR/stimulus-R interval until the return of the positive T wave to the isoelectric baseline (Zhang et al., 2014). Corrected QT (QTc) interval was measured using Bazett’s formula normalized as QTc = QT/(RR/f)1/2, in which RR is the R–R interval and f = 150 ms for rats and f = 100 ms for mice according to Mitchell et al. (1998) and Kmecova and Klimas (2010), respectively.

Ventricular effective refractory period was evaluated using a pacing MBHE implanted on the left ventricle. VERP was determined as the longest S1–S2 interval that failed to generate a response. A drive train of 20 S1 at various CL was used in this protocol followed by an S2 which evaluated the VERP in the millisecond range. The VERP was determined by three consecutive S1–S2 drive trains which failed to induce ventricular capture following the S2.

### LV MAP Recordings in Isolated-Perfuse Mice Hearts

Mice were heparinized (500 U kg^-1^, IP) and anesthetized (ketamine/xylazine 75/5 mg/kg^-1^ IP). Hearts were rapidly excised, washed in ice-cold oxygenated Tyrode’s solution containing (mM): NaCl 118, KCl 5.4, CaCl_2_ 2.5, MgCl_2_ 0.5, NaH_2_PO_4_ 0.39, glucose 11, Hepes 10 titrated to pH 7.4, and cannulated via the aorta. Thereafter, the hearts were perfused using a constant coronary perfusion pressure of 100 mmHg with Tyrode’s solution pre-heated to 37°C. For technical reasons, we could not use a heating cup covering the heart during the MAP measurements. Therefore, the epicardial surface of the heart had a temperature lower than 37°C (∼33°C). The experiment began after 30 min of stabilization. MAP recordings from the surface of the LV were performed using an Ag–AgCl silver wire inserted into a polyethylene tube (1 mm in diameter). The tip of the polyethylene tube was in contact with the heart. The ground electrode was an Ag–AgCl silver wire fastened outside the tube several millimeters above the tip. The polyethylene tubing was positioned on the heart so that the central silver wire made contact with the epicardial surface of the ventricle. LV MAP measurements were obtained when the polyethylene tube made contact with the epicardial surface of the LV and mild pressure was applied. MAP recordings were first obtained in each animal during sinus rhythm and then pacing at double diastolic threshold was applied using an MBHE positioned on the RV. During preliminary experiments, we found that excessive rapid pacing of the ventricle, which was require to determine the VERP, usually induced long-term shortening of the APD or instability of the MAP recordings. Thus, in order to prevent changes in MAP and VERP due to excessive rapid pacing we measured and compared MAP and VERP as a function of BCL using 10 beat S1S2 drive trains at 150 ms BCL vs. 100 ms BCL only. In addition, as previously reported (Knollmann et al., 2007) higher pacing rates markedly attenuated the fast peak of the MAP signal (**Figures 5A,C**). Therefore, in order be able to directly compare the late phase of the action potential in both BCLs, APD90 values were measured relative to the action potential amplitude recorded at 150 ms BCL.

### Statistical Analysis

Values are expressed as mean ± SE. Paired *t*-test or one-way ANOVA of repeated measures and Tukey multiple comparisons post-test were used as required. The criterion for significance was set at *p* < 0.05. Unless otherwise stated *p*-values are displayed graphically as follows: ^∗^*p* < 0.05, ^∗∗^*p* < 0.01, ns = not significant.

## Results

**FIGURE 2:**
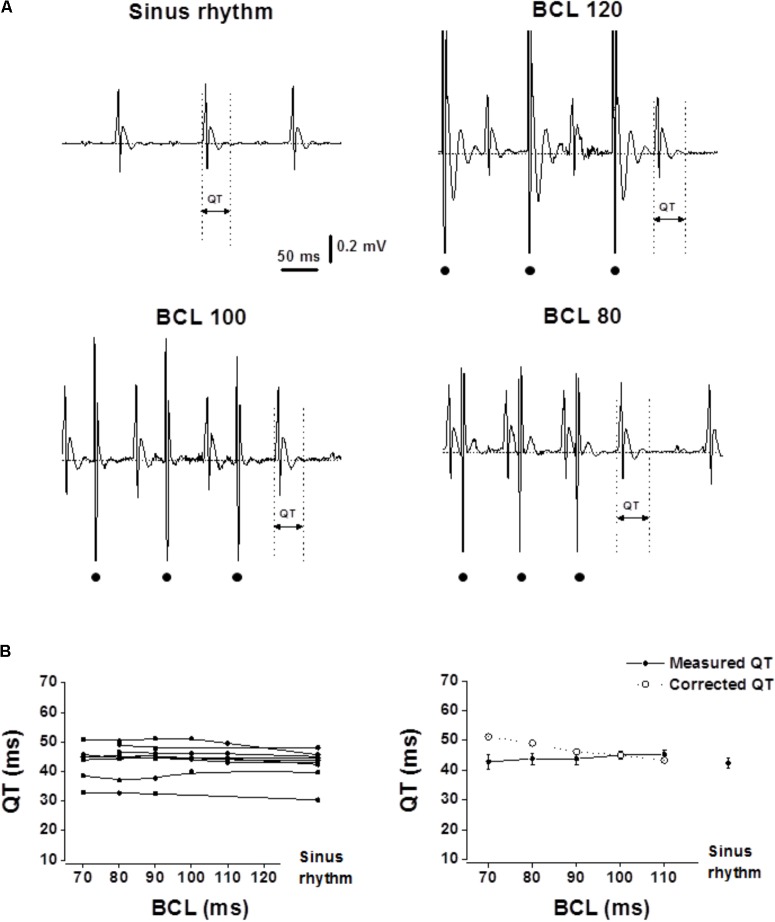
Measured QT intervals in unanesthetized mice show flat rate dependence. Analysis of the QT interval in mice following atrial pacing at various BCLs. **(A)** Example of ECG recordings at sinus rhythm and during atrial pacing at various BCLs (120, 100, and 80). Dots at the bottom indicate atrial pacing. **(B)** Left: QT interval measurements of individual mice as a function of BCL (*n* = 9). Right: Averages of measured QT and calculated QTc plotted as a function of BCL. Statistical analysis did not reveal any significant changes in QT measurement as a function of BCL. For detailed data of individual mice see **Supplementary Table S1**.

Following the 6 day recovery period all animals participating in the study were well-recovered and gained their pre-operative weight. High-quality recordings were obtained throughout the pacing protocol period for each of the animals (**Figures 1A, 2A**). Recordings were all obtained during the early phase of light in the animal facility rooms, a period when the rodents mostly sleep and have reduced motor activity. During occasional periods of movement which induced muscle and motion artifacts in the ECG, analysis was paused until the animal was resting again.

### QT Measurements

In both rats and mice, the QT interval was initially acquired during normal sinus rhythm. Next, drive trains with different basic cycle length (BCL) were applied starting from near normal BCL and reducing the BCL in 10 ms steps until there was a loss of 1:1 capture in the ventricular myocardium. In both species, the measured QT interval was similar in all atrial pacing BCLs (**Figure 1**, range: 160–80 ms for rats. **Figure 2**, range: 120–70 ms for mice). Calculating the corrected QT interval according to the formulas suggested by Kmecova and Klimas (2010) for rats and Mitchell et al. (1998) for mice resulted in marked difference between the measured QT and the corrected QT for a wide range of BCLs in both species (**Figures 1, 2**). These results strongly support the notion that correction of the QT for HR is not required in rodents (see section “Discussion”).

### VERP Measurements

**FIGURE 3:**
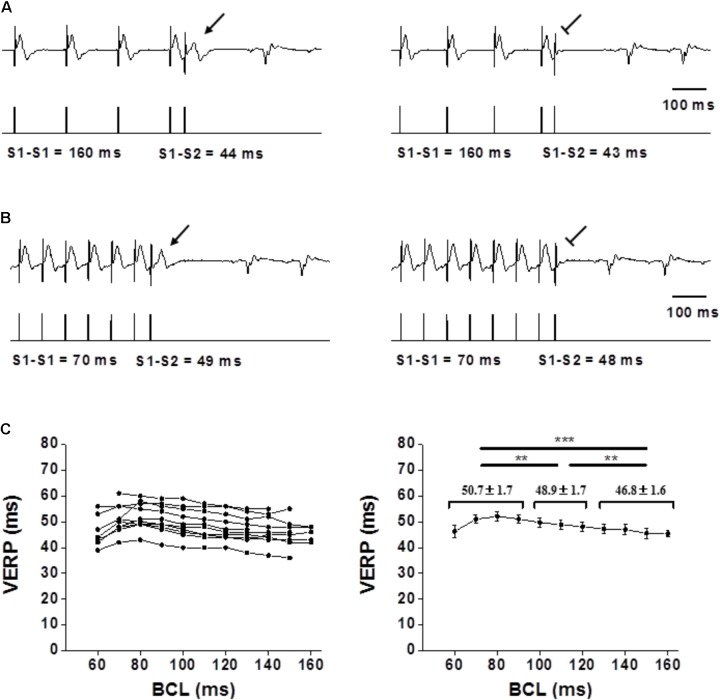
Flat VERP rate dependence in unanesthetized rats. **(A,B)** Examples of VERP determination at a 160-ms BCL and 70-ms BCL, respectively. The upper traces denote ECG recording, and the lower traces mark the pacing through the ventricular electrode. Note the absence of VERP shortening in **(B)**. **(C)** Left: VERP as a function of BCL for each individual rat (*n* = 10). Right: Average VERP measurements for all rats. Pooled data analysis showed slight but significant prolongation of the VERP as the BCL decreased Means ± SE are shown for the pooled data of BCL ≥ 130 ms, BCL between 120 and 100 ms and BCL < 100 ms. For detailed data of individual rats see **Supplementary Table S1**.

**FIGURE 4:**
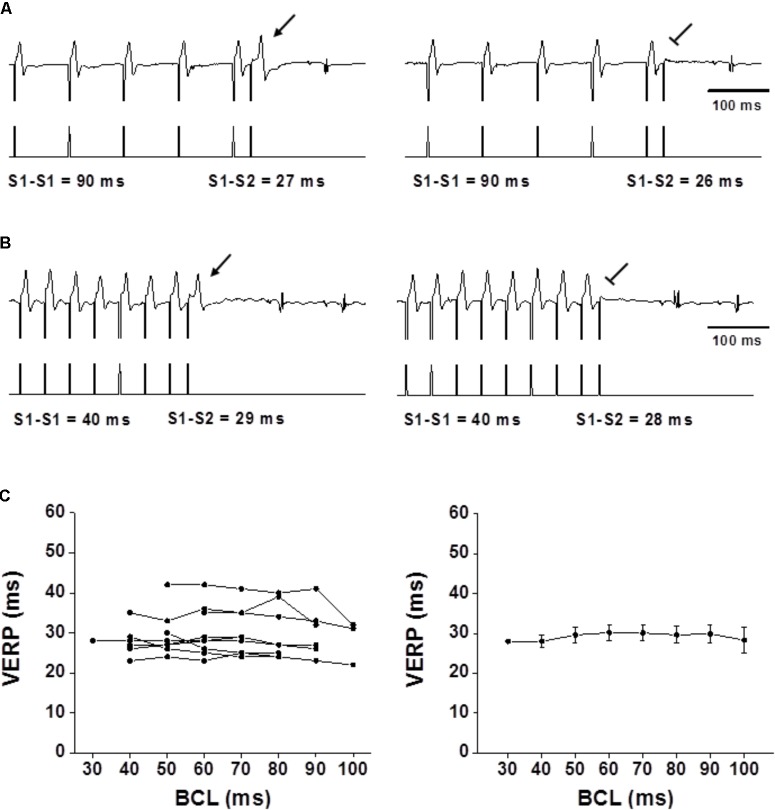
Flat VERP rate dependence in unanesthetized mice. **(A,B)** Examples of VERP determination at a 90-ms BCL and 40-ms BCL, respectively. The upper traces denote ECG recording, and the lower traces mark the pacing through the ventricular electrode. Note the absence of VERP shortening in **(B)**. **(C)** Left: VERP as a function of BCL for each individual mouse (*n* = 9). Right: Averaged VERP measurements for all mice. Statistical analysis did not find any significant changes between the VERP measurements in the different BCLs with and without data grouping as in **Figure 3**. For detailed data of individual rats see **Supplementary Table S1**.

The refractoriness of the myocardium is strongly affected by the APD with a recovery of excitability occurring at ∼85% repolarization over a wide range of steady-state CLs (Franz et al., 1990; Koller et al., 1995). Typical rate-adaptation in which APD and ERP becomes shorter as BCL decreases is a consistent finding in large mammals (Franz et al., 1988). In the rodent myocardium, typical rate-adaptation of atrial and ventricular APD has been documented in the *ex vivo* mouse heart (Knollmann et al., 2007; Waldeyer et al., 2009), but reports in *ex vivo* and anesthetized preparations indicate absence of ERP rate-adaptation (Etzion et al., 2008; Waldeyer et al., 2009). Our current measurements in the freely moving state demonstrate total absence of typical VERP rate-adaptation in both rats and mice (**Figures 3, 4**, respectively). It is noteworthy that while in the mice the curve was totally flat, some reverse-adaptation characterized by increased VERP in the presence of shorter BCLs was observed in the rats. However, this effect, although significant is of rather modest magnitude (**Figure 3C**).

### Combined VERP and MAP Recordings in Isolated Mice Hearts

**FIGURE 5:**
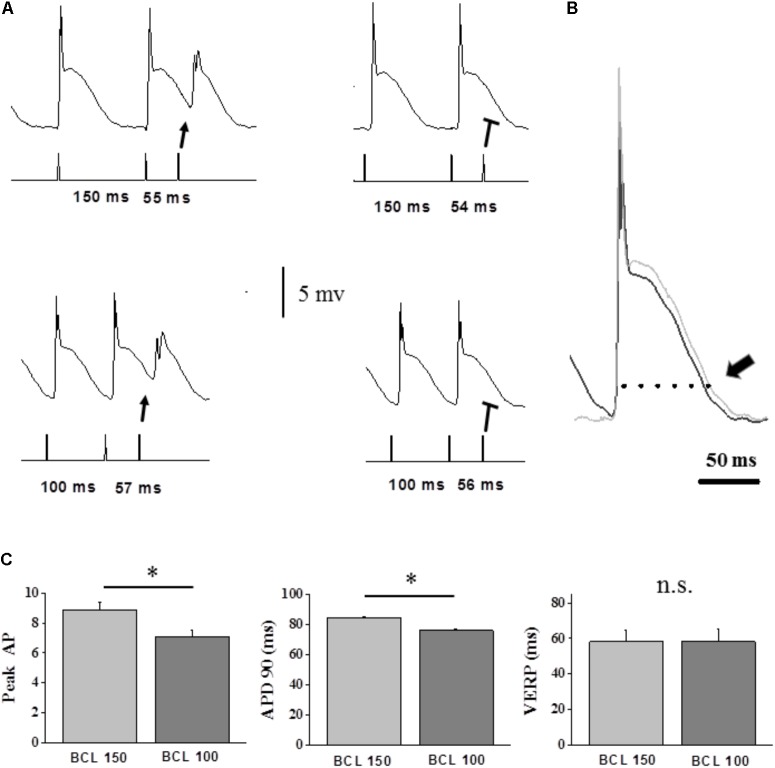
Dispersion between VERP and MAP recordings as a function of BCL in the isolated-perfused mouse hearts. **(A)** Example of simultaneous APD and VERP measurements using 10 beats S1S2 drive train. Left traces indicate the shortest BCLs for which S2 generates a response. Upper recordings: traces at 150 ms BCL indicating a VERP of 54 ms. Lower recordings: traces at 100 ms BCL indicating a VERP of 56 ms. This mild VERP prolongation (confirmed three times) occurred despite a sight shortening of the APD90. Note however, that the average VERP of all the preparations was not statistically different between the two BCLs as shown in **(C)** below. **(B)** Superimposed high magnification traces from **(A)**, demonstrating the MAP recorded action potentials at 150 ms BCL (black) and 100 ms BCL (light gray). Arrow marks the APD_90_ difference. Note that VERP did not follow this slight shortening in the APD the even slightly increased to 56 ms. **(C)** Analysis of VERP and MAP rate-dependence in isolated-perfused mice hearts (*n* = 5). Comparison of parameters during 10 beats S1S2 drive train at 150 ms BCL vs. 100 ms. VERP measurements did not show any differences between the values in both BCLs. The APD and Peak AP values are averages of the seven last S1-derived action potentials in each train. ^∗^*p* < 0.05.

To directly compare APD and VERP rate-dependence, we evaluated both parameters simultaneously under *ex vivo* conditions in the mice heart. As explained in Section “Materials and Methods,” technical limitations constrained our analysis to comparisons between two different BCLs only (150 vs. 100 ms). As reported previously (Knollmann et al., 2007; Waldeyer et al., 2009) we observed shortening the APD_90_ following pacing using 100 ms CL as compared to 150 ms (**Figure 5**). However, this shortening was not translated into shorter VERP indicating that at high frequencies the VERP is not entirely dependent on APD and there is∖are other factor∖s which induce dissociation between the APD and the VERP. Quantitative analysis of the MAP vs. VERP measurements is shown in **Figure 5C**.

## Discussion

This study evaluated the rate-dependent characteristics of the QT interval and the VERP in unanesthetized freely moving rats and mice. In order to utilize rodents appropriately in pacing and arrhythmia research, understanding of their native cardiac electrophysiology is necessary. However, up to now such studies were mainly performed *ex vivo* or in anesthetized open-chest or closed-chest preparations (Choy et al., 2016; Yaegashi et al., 2016; Huang, 2017). Cardiac electrophysiology in such preparations may not represent the normal properties as in the freely moving state. Moreover, neither of these approaches are amenable to longitudinal examination, making it impossible to follow progressive changes in electrophysiological parameters as we recently reported with the present technology in rats subjected to different ventricular pacing modes (Mulla et al., 2017).

The main finding of the current study is that awake rodents do not have typical rate-adaptation of their QT interval or VERP. We found that QT values were stable over a wide range of atrial pacing frequencies (160–80 ms BCL in rats and 120–70 ms BCL in mice). Accordingly, the VERP values did not decrease in the presence of shorter BCLs in both species. Thus, in practical terms our data indicate no physiological basis for QT correction in rodent studies. In large mammals, the APD becomes shorter with decreasing CLs allowing for a longer diastolic period. Block of the heart beats and skipped contractions could occur if the heart did not adapt to a significant rate increase by shortening of the APD. However, the cardiac response of small rodents is markedly different in this regard. While humans can increase their HR during exercise by 200%, rats and mice can increase their HRs only by approximately 40–50 and 30–40%, respectively (Lujan et al., 2012). Thus, the rate-adaptation difference between large mammals and rodents seems consistent with their different physiological needs. One limitation that should be mentioned regarding the QT interval findings of this study relates to use of a single peripheral recording lead: since the spatio-temporal morphology of the ECG is the result of the motion of the cardiac extracellular action potentials with respect to the position of the electrodes, we cannot exclude the possibility that by using a multi-lead approach that would displays a more detailed view of the cardiac electrical field, some additional findings would be noted. Nevertheless, the fact that the QT recordings did not show rate-dependence in any of the tested animals of this study (10 rats and 9 mice), while our pseudo-ECG did have some variability in leads location, support the notion that the observed rate insensitivity of the QT interval is indeed a valid finding. A multi-lead approach requires some technical adaptations of the current system, but should be considered in future studies of this issue.

Studies describing the APD restitution curve in healthy rat preparations, whether in whole hearts by MAP recordings and optical mapping or in isolated myocytes, showed almost flat APD restitution curves (Benoist et al., 2011, 2012). Our current findings in unanesthetized rats support the relevance of these findings in terms of normal rat physiology. In contrast, in mice the atrial and ventricular APD_90_ restitution curves were reported to exhibit typical rate-dependence, which was less clear than in large mammals but was still prominent (Knollmann et al., 2007; Waldeyer et al., 2009). Importantly, in Waldeyer et al. (2009) the same *ex vivo* preparations in which APD was reported to have rate-adaptation did not show VERP rate dependence. In addition, our group which was the first to analyze in details the *in vivo* rate-dependence of atrial ERP in rats and mice, demonstrated totally flat rate-adaptation curves in both species (Etzion et al., 2008). Thus, previous and current data strongly support the notion that the reported APD rate-adaptation of the mice myocardium do not affect as expected the ERP and QT interval of these rodents.

One possibility that can partially explain the discrepancy between the APD rate-adaptation and the flat VERP rate-dependence in the mice heart is a rate dependent dissociation between the APD and the ERP at high firing frequencies. To explore this possibility, we performed *ex vivo* experiments combining MAP recording and VERP measurements. Although technical limitations constrained our analysis to only two rather long BCLs, our findings support the notion that such a dissociation exists at high pacing rates. It is conceivable that a prominent contributing factor to this phenomenon is the reduced recovery of fast Na^+^ channels from inactivation at higher pacing rates. The marked effect of higher pacing rates on the amplitude of the fast component of the MAP signal can support this notion. Such an effect can lead to decreased availability of Na^+^ channels for the next action potential initiation causing a post-repolarization refractoriness effect. A small increase in the resting membrane potential can be a contributing factor as well. The latter is analogous to the effect of ischemia on mammalian hearts, where a depolarization of the resting membrane potential results in a post-repolarization refractoriness (Burton and Cobbe, 2001). It will be important to further validate our current *ex vivo* finding using more physiologic pacing rates, which have led to preparation instability in the current setting. The use of blood-perfused preparation or alternatively the application of an electromechanical uncoupler that will prevent energy exhaustion may help in overcoming the rate limitation we encountered.

While the above data can, at least partially, explain the dissociation between APD and VERP in terms of rate-dependence, it does not explain a similar dissociation between APD and QT interval in mice. It is possible that the observed APD rate-adaptation of the mouse heart does not manifest itself *in vivo.* In addition, mice QT is so short that significant repolarization already occurs before ventricular activation has completed in different parts of the ventricles (Curtis et al., 1987; Boukens et al., 2012). Thus, it is possible that the APD shortening at high pacing rates is counterbalanced by a prolonged ventricular activation time due to increased Na^+^ channels inactivation, leading to a net zero effect. Further understanding of this issue may be markedly advanced by simultaneous measurements of QT interval and ventricular APD *in vivo*.

## Author Contributions

WM: contributed to the conception and design of the study, performed experiments, performed the statistical analysis, designed the figures, and wrote the first draft of the manuscript. RG: performed experiments, analyzed the data. MMo: performed experiments, analyzed the data. MMu, HK-G, and SE: performed experiments. NL-C and OB: critically revised the MS. YE: conceived and designed the study, guided the final analysis and design of the figures, wrote the final version of the MS. All authors contributed to manuscript revision, read and approved the submitted version.

## Conflict of Interest Statement

The authors declare that the research was conducted in the absence of any commercial or financial relationships that could be construed as a potential conflict of interest.

## Supplementary Material

TABLE S1 QT and VERP data of individual animals.
